# Structural Aspects of Antioxidant and Genotoxic Activities of Two Flavonoids Obtained from Ethanolic Extract of* Combretum leprosum*

**DOI:** 10.1155/2016/9849134

**Published:** 2016-07-05

**Authors:** Cassiana Macagnan Viau, Dinara Jaqueline Moura, Pricila Pflüger, Valdir Alves Facundo, Jenifer Saffi

**Affiliations:** ^1^Department of Basic Health Sciences, Laboratory of Genetic Toxicology, Federal University of Health Sciences of Porto Alegre (UFCSPA), Rua Sarmento Leite 245, 90150-170 Porto Alegre, RS, Brazil; ^2^National Institute for Translational Research on Health and Environment in the Amazon Region, CNPq/INCT/INPeTAm, Rio de Janeiro, RJ, Brazil; ^3^Department of Pharmacosciences, Laboratory of Genetic Toxicology, UFCSPA, Rua Sarmento Leite 245, 90150-170 Porto Alegre, RS, Brazil; ^4^Núcleo de Ciência e Tecnologia, Universidade Federal de Rondônia, Avenida Presidente Dutra, Até 2965, lado Ímpar, Centro, 76801059 Porto Velho, RO, Brazil

## Abstract

*Combretum leprosum* Mart., a member of the Combretaceae family, is a traditionally used Brazilian medicinal plant, although no evidence in the literature substantiates its antioxidant action and the safety of its use. We evaluated the antioxidant properties of the ethanolic extract (EE) from flowers of* C. leprosum* and its isolated products 5,3′-dihydroxy-3,7,4′-trimethoxyflavone (FCL2) and 5,3′,4′-trihydroxy-3,7-dimethoxyflavone (FCL5) in* Saccharomyces cerevisiae* strains proficient and deficient in antioxidant defenses. Their mutagenic activity was also assayed in* S. cerevisiae,* whereas cytotoxic and genotoxic properties were evaluated by MTT and Comet Assays, respectively, in V79 cells. We show that the EE, FCL2, and FCL5 have a significant protective effect against H_2_O_2_. FCL2 showed a better antioxidant action, which can be related to the activation of the 3′-OH in the presence of a methoxyl group at 4′ position in the B-ring of the molecule, while flavonoids did not induce mutagenesis in yeast, and the EE was mutagenic at high concentrations. The toxicity of these compounds in V79 cells increases from FCL2 = FCL5 < EE; although not cytotoxic, FCL5 induced an increase in DNA damage. The antioxidant effect, along with the lower toxicity and the absence of genotoxicity, suggests that FCL2 could be suitable for pharmacological use.

## 1. Introduction

Reactive oxygen species (ROS) and reactive nitrogen species (RNS) are responsible for the oxidative stress that can lead to physiopathological processes such as aging, atherosclerosis, inflammation, and Alzheimer's and Parkinson's diseases and are related to the etiology of several cancers. In recent years, there has been steady interest in finding solutions to avoid the formation of these reactive species or to prevent their action in the cell [1–3]. Living organisms possess numerous antioxidant defenses and repair mechanisms against oxidative stress. However, these mechanisms sometimes are not sufficient to prevent the damage, which can result in tissue damage and loss of function in a number of tissues and organs [4].

Natural products derived from plants have been widely studied due to their great pharmacological potential [5]. The medicinal use of plants of the Combretaceae family is widely described in the scientific literature [6–9]. This family is distributed in 20 genera with 600 species. The largest genera are* Combretum* and* Terminalia*, with around 370 and 200 species, respectively [10]. Members of Combretaceae occur mainly in tropical and subtropical areas, including Africa and Brazil [11].

**Figure 1:**
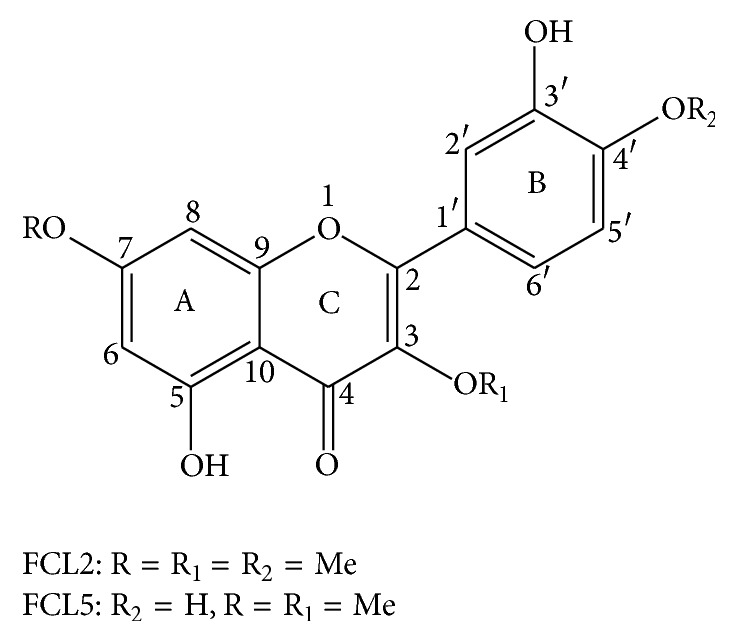
Chemical structures of FCL2 (5,3′-dihydroxy-3,7,4′-trimethoxyflavone) and FCL5 (5,3′,4′-trihydroxy-3,7-dimethoxyflavone) products isolated from the flowers of* Combretum leprosum*.

Species of this genus are found in North and Northeastern Brazil, namely,* Combretum leprosum* Mart., popularly known as mofumbo, mufumbo, or cipoaba in Brazil. Infusions prepared with the aerial parts (stems, leaves, and flowers) and roots of* C. leprosum* are used in folk medicine to heal wounds, to treat hemorrhages, or as a sedative [12, 13]. According to phytochemical analysis,* C. leprosum* is rich in compounds such as cycloartanes, triterpenes (arjunolic and mollic acid and 3*β*,6*β*,16*β*-trihydroxy-lup-20(29)-ene), and flavonoids (3-O-methylquercetin, 5,3′-dihydroxy-3,7,4′-trimethoxyflavone (FCL2, Figure 1), 5,3′,4′-trihydroxy-3,7-dimethoxyflavone (FCL5, Figure 1), and quercetin) and some of these substances have proven biological activity [12, 14–18]. More recently, our research group showed that the pentacyclic triterpene 3*β*,6*β*,16*β*-trihydroxylup-20(29)-ene (TTHL) has a potent antiproliferative activity in MCF-7 breast cancer cells [19]. The ROS formation by TTHL and its direct interaction with DNA indicated that treating MCF-7 cells with TTHL causes cascade signaling in the induction of caspases, which in turn governs the mechanisms for inducing apoptosis [19].

Taking into account the popular use of* C. leprosum* and its previously described pharmacological activities, this work aims to increase the knowledge about this species by evaluating antioxidant, cytotoxic, and mutagenic/genotoxic activities of the ethanolic extract (EE) and its compounds FCL2 and FCL5, in the yeast* Saccharomyces cerevisiae* and in V79 mammalian cells.

## 2. Materials and Methods

### 2.1. Chemicals

Dulbecco's modified Eagle's medium (DMEM), low-melting-point agarose (LMP), high-melting-point agarose (HMP), phosphate-buffered saline (PBS), and hydrogen peroxide (H_2_O_2_), amino acids, and nitrogen bases were purchased from Sigma (St. Louis, MO, USA). Foetal bovine serum (FBS) and penicillin/streptomycin were obtained from Gibco-BRL (Grand Island, NY, USA). MMS (methyl methanesulfonate) was purchased from Sigma (St. Louis, MO, USA). Yeast extract, bacto-peptone, bacto-agar, and yeast nitrogen base were obtained from Difco Laboratories (Detroit, MI). All other chemicals were of the highest purity grade commercially available.

### 2.2. Plant Material

Botanical material was collected by Dr. Edilberto Rocha Silveira (Federal University of Ceará, Fortaleza) in May 2007 in a free area of Viçosa, Ceará State, Brazil, and was identified by Dr. Afrânio Fernandes (Federal University of Ceará, Fortaleza) as* Combretum leprosum* Mart. A voucher specimen was deposited in the Herbarium Prisco Bezerra of the Biology Department, Federal University of Ceará, Brazil, under number 12446. All necessary permits were obtained for the harvesting of the flowers.

### 2.3. Ethanolic Extract (EE) Obtention and FCL2 and FCL5 Isolation and Purification

The dried flowers (2.7 kg) were powdered and extracted with ethanol (5 L), being stirred and macerated at room temperature (24 ± 3°C) for approximately 24 h. This procedure was repeated three times. The solvent was fully evaporated under reduced pressure and the EE (yield 58.3 g) was lyophilized and stored in a freezer at −20°C until use. Part of the EE (30.0 g) was subjected to column chromatography on silica gel, eluted with n-hexane, ethyl acetate (EtOAc), or methanol (MeOH). The EtOAC fraction was further purified by column chromatography over silica gel using gradient elution with n-hexane-EtOAc mixtures to obtain FCL2 (12.1 mg; 60 : 40) and FCL5 (19 mg; 75 : 25). The chemical structures of the isolated products were established based on their spectral data and by comparison with those reported in the literature (for more details see [20]).

For cell treatments, stock solutions of EE, FCL2, and FCL5 were prepared immediately prior to use, with dimethyl sulfoxide (DMSO) as solvent. The appropriate concentrations were obtained by diluting the stock solution in sterile distilled water, and the final concentration of DMSO in the incubation mixture never exceeded 0.1%. Control samples were always treated with the same amount of DMSO (0.1% v/v) used in the corresponding experiments.

### 2.4. Assays with* S. cerevisiae*

#### 2.4.1. Strains, Media, and Treatment

Media, solutions, and buffers were prepared according to Burke et al., 2000 [21]. YPD medium (0.5% yeast extract, 2% peptone, and 2% glucose) was used for routine growth. Synthetic complete medium (SC), 0.67% yeast nitrogen base, 0.1% ammonium sulfate, and 2% glucose, supplemented with the appropriate amino acids and bases (40 mg/mL) was used for the detection of mutations.

Stationary phase cultures were obtained by inoculation of a single colony onto liquid YPD. We chose to work in the stationary phase of growth because this resembles most cells of multicellular organisms in important aspects: (i) most energy comes from mitochondrial respiration; (ii) the cells have left the active cell cycle and have entered the G_0_ phase; and (iii) damage accumulates over time [22, 23].

#### 2.4.2. Antioxidant Assay

WT cells and isogenic mutant strains of* S. cerevisiae* lacking antioxidant defenses (Table 1) in stationary phase were treated with several concentrations of the EE, FCL2, and FCL5, at concentrations of 10, 50, 100, and 500 *μ*g/mL, for 1 h at 30°C. To verify an intracellular protective effect of EE, FCL2, and FCL5, yeast cells were washed and then exposed to a sublethal concentration of H_2_O_2_ (5 mM) in PBS for another hour. Suitable aliquots were plated in triplicate on solid YPD (2-3 days, 30°C) and colony-forming units were counted. Sensitivity was expressed as percentage of survival in relation to the negative control (solvent) [24].

#### 2.4.3. Mutagenic Assay

The detection of reverse and frameshift mutations was performed using the XV185-14c haploid strain (Table 1). Cell cultures were grown as described above, exposed to EE, FCL2, and FCL5 at concentrations of 10, 50, 100, and 500 *μ*g/mL, and then incubated in PBS for 1 h at 30°C. Two alleles, his1-798 and lys1-1, were used to detect point mutagenesis. The suppressible ochre nonsense mutant allele lys1-1 can be reverted either by locus-specific sequence alteration (true reversion) or by a forward mutation in a suppressor gene. Distinction between true reversions and forward (suppressor) mutations at the lys1-1 locus was according to Schuller and von Borstel [25], where the reduced adenine content of the SC-lys medium shows true reversions as red and suppressor mutations as white colonies. Survival was determined on SC (3–5 days, 30°C) and mutation induction (HIS, LYS, or HOM revertants) on media lacking the appropriate amino acid (7–10 days, 30°C).

### 2.5. Assays with Mammalian V79 Cells

#### 2.5.1. Culture and Treatment

Chinese hamster lung fibroblast (V79) cells were cultured under standard conditions in DMEM. Cells (5 × 10^5^ cells/mL) were seeded in complete media and grown for 1 day prior to treatment with EE and isolated products (FCL2 and FCL5) before evaluation by MTT and Comet Assays. The EE and isolated products were added to FBS-free media to achieve concentrations from 10, 25, 50, and 75 *μ*g/mL. Cells were treated for 3 h under standard conditions. MMS (40 *μ*M) was used as the positive control.

#### 2.5.2. Cell Viability Assay

MTT reduction was performed according to Denizot and Lang [26]. Briefly, after the treatments, cells were washed once with PBS before the addition of 100 *μ*L of serum-free media containing yellow tetrazolium salt (MTT; 1 mg/mL) dye. After 3 h of incubation at 37°C, the supernatant was removed, and the residual purple formazan product was solubilized in 200 *μ*L DMSO, stirred for 15 min, and its absorbance was measured in a SpectraMax reader (Bio-Rad, USA) at a wavelength of 570 nm. The absorbance of the negative control was set as 100% viability, and the values for treated cells were calculated as a percentage of the control.

#### 2.5.3. Alkaline Comet Assay

The alkaline Comet Assay was performed as described by Singh et al. [27]. Briefly, 10 *μ*L of cell suspension (10,000 cells) treated with the EE or the isolated products (FCL2 and FCL5) was mixed with 90 *μ*L LMP agarose, spread on a normal agarose precoated microscope slide, and placed at 4°C for 5 min to allow for solidification. Cells were lysed in high salt and detergent (2.5 M NaCl, 100 mM Na_2_EDTA, 10 mM Tris with 1% Triton X-100, and 10% DMSO freshly added) for 2 h. Slides were removed from lysing solution and washed three times in PBS. Subsequently, cells were exposed to alkali conditions (300 mM NaOH/1 mM Na_2_EDTA, pH > 13, 30 min, 4°C) to allow DNA unwinding and expression of alkali-labile sites. Electrophoresis was conducted for 25 min at 25 V and 300 mA (94 V/cm). After electrophoresis, the slides were neutralized and silver stained [28]. One hundred cells were visually scored according to the tail length and the amount of DNA present in the tail. Each comet was given an arbitrary value of 0–4 (0, undamaged; 4, maximally damaged), as described by Collins et al. [29]. A damage score was thus assigned to each sample and can range from 0 (completely undamaged: 100 cells × 0) to 400 (with maximum damage: 100 cells × 4). International guidelines and recommendations for the Comet Assay consider visual scoring of comets a well-validated evaluation method since it is highly correlated with computer-based image analysis [29, 30].

### 2.6. Statistical Analysis

All experiments were independently repeated at least three times. Results are expressed as means ± standard deviation (SD). Data were analyzed by one-way analysis of variance (ANOVA), and means were compared using Tukey test, with *P* ≤ 0.05 considered as statistically significant.

## 3. Results and Discussion

In contrast with conventional drugs research and development, the toxicity and genotoxicity of traditional herbal medicines are not often evaluated [18]. Most of the population, however, believes that if these products have been used so far, they should be devoid of toxicity. For this reason, an assessment of cytotoxic and genotoxic potentials is necessary to ensure the relatively safe use of* C. leprosum*. In the present study, different assays were performed to reveal whether the EE from flowers of* C. leprosum* and its isolated products FCL2 and FCL5 present antioxidant, mutagenic, and/or genotoxic properties.

The beneficial health effects of polyphenol-rich plants are often attributed to their potent* in vitro* antioxidant activities, since diets rich in polyphenols are epidemiologically associated with a decrease in the incidence of age-related diseases in humans [31]. However, medicinal plants may also exert prooxidant effects that upregulate endogenous protective enzymes [32, 33]. ROS attack almost all cell components, including DNA, proteins, and lipid membranes, and therefore are able to cause lethal damage to cells [34]. Furthermore, ROS toxicity has been implicated in a variety of human diseases and in the aging process, as well as in the multiple-stage events of carcinogenesis [35]. To investigate the antioxidant effect of the studied products in living systems, we used H_2_O_2_ to induce oxidative damage in* S. cerevisiae* strains defective in several antioxidant defenses.* S. cerevisiae* has been a useful model to study the eukaryotic response to oxidant challenge and to investigate the interplay between oxidative stress resistance and level of damaged cell components such as DNA [36]. It produces a variety of enzymes, such as superoxide dismutase (SOD), catalase, and glutathione peroxidase, and small molecules and peptides (glutathione and thioredoxins), which detoxify ROS [37, 38]. In our study, the EE showed a protective effect against oxidative stress induced by H_2_O_2_, indicating antioxidant properties (Table 2). The antioxidant activity of the ethanolic extracts from leaves of* Combretum decandrum* and* Combretum duarteanum* was previously demonstrated* in vitro* by thiobarbituric acid reactive species (TBARS), hydroxyl radical-scavenging, and scavenging activity of nitric oxide assays [39, 40].

Superoxide anion has been shown to inactivate certain (4Fe-4S) cluster-containing enzymes by oxidizing one iron atom, thereby causing its release from the cluster [41]. This process leads to both enzyme inactivation and further oxidative damage of cellular components, since free iron can promote the formation of ^•^OH radical via Fenton reaction [35]. The mutants, in the absence of SOD, can accumulate excess of O_2_^•−^ anion and free iron as a result [42]. In this case,* C. leprosum* is probably acting as a chemical defense against superoxide radical in strains without the specific enzymes that convert this radical to a less reactive H_2_O_2_ molecule.

Moreover, our findings showed that FCL2 was noncytotoxic at the concentrations tested and significantly enhanced (at 10–500 *μ*g/mL) the survival of mutant yeast cells upon H_2_O_2_ exposure (Table 3). FCL5 also induced significant increase of survival in oxidative defense deficient yeast cells at the range of 10–500 *μ*g/mL (Table 4). The antioxidant activity of both FCL2 and FCL5 flavonoids can be due to the presence of the 2,3 double bond in conjugation with a 4-oxo function in the C-ring. This is responsible for electron delocalization from the B-ring in these molecules. As previously described [43], the antioxidant potency is related to structure in terms of electron delocalization of the aromatic nucleus, where these compounds react with free radicals, and phenoxyl radicals produced are stabilized by the resonance effect of the aromatic nucleus. Accumulating evidence suggests that the 5-OH group with 4-oxo functions in A-ring and C-ring equally contribute to a maximum radical scavenging potential [31]. Regarding FCL2 and FCL5, both flavonoids have a hydroxyl group in C-5 in the A-ring (Figure 1). As these compounds have identical chemical structure (except for* ortho* position in the B-ring), the structure-activity relationship (SAR) studies of FCL2 and FCL5 flavonoids showed that the antioxidant effect occurs as follows: 4′-Me > 4′-OH = 3′-OH = C2-C3 double bound = 5-OH = 3-Me = 7-Me (Figure 1).

In the double mutant* sod1*Δ*sod2*Δ, FCL2 seems to be more protective than FCL5 and EE (Tables 2 [Table tab3]–4). The individual difference between FCL2 and FCL5 products is the presence of a methyl group at position C4′ in the B-ring of FCL2 (Figure 1). In general, the “classical” antioxidant nature of flavonoids is defined mainly by the presence of a B-ring catechol group (dihydroxylated B-ring), which is capable of readily donating hydrogen (electron) to stabilize a radical species [44, 45]. Nevertheless, despite the presence of 4-methoxy substitution in the B-ring, the FCL2 methylated flavonoid still behaved as a better antioxidant than 3′,4′-dihydroxy FCL5 flavonoid (Table 2). A possible explanation for the FCL2 methylated flavonoid having a better antioxidant activity than 3′,4′-dihydroxy FCL5 flavonoid can be related to activation of the 3′-OH in the presence of a methoxyl group at 4′ position. To investigate this underlying molecular mechanism, Van Acker et al. [46] tested a large group of flavonoids from all major structural subclasses and their ability to chelate iron and avoid lipid peroxidation. They concluded that an* O*Me or* O*EtOH substituent on the 4′ position in the B-ring could activate the 3′-OH, as shown for hesperetin and hesperidin, which are much more active than the 4′-OH compounds naringin, naringenin, and apigenin.

The results obtained by Dueñas et al. [47] showed that* O*-methylated quercetin, catechin, and epicatechin metabolites still retain significant radical scavenging activity at pH 7.4, suggesting that they could act as potential antioxidants in physiological conditions. It was confirmed that the antioxidant activity of these flavonoids strongly depends on the pH of the medium, with high activity in physiological conditions. We propose here that the increase in radical scavenging activity of FCL2 flavonoid upon methylation of the catechol moiety can be explained by the activation of the 3′-OH in the presence of a methoxyl group at 4′ position and higher levels of deprotonation at physiological conditions. The* O*-methylation could affect the electronic properties (especially of deprotonated forms) increasing their ability to donate electron and hydrogen atoms [for more details and comparison of similar structures of flavonoids, see Van Acker et al. [46] and Dueñas et al. [47]].

Both FCL2 and FCL5 flavonoids did not induce either cytotoxicity or mutations in the XV185-14c yeast strain (Tables 6 and 7), whereas the EE was clearly mutagenic in point reversion assay for the* his1-798* mutant allele [*F*(5.84) = 29.76; *P* < 0.05] and ochre* lys1-1* mutant allele [*F*(5.44) = 23.89; *P* < 0.05] in haploid strain XV185-14c at the highest concentration (500 *μ*g/mL) with a low level of toxicity (12%) (Table 5). The mutagenic effect of the EE could be related to the presence of flavanone compounds (naringenin, pinocembrin, and eriodictyol) found in* Combretum* species [9] which despite their high antioxidant activity showed toxicity and genotoxicity in different biological models [48–52].

**Figure 2:**
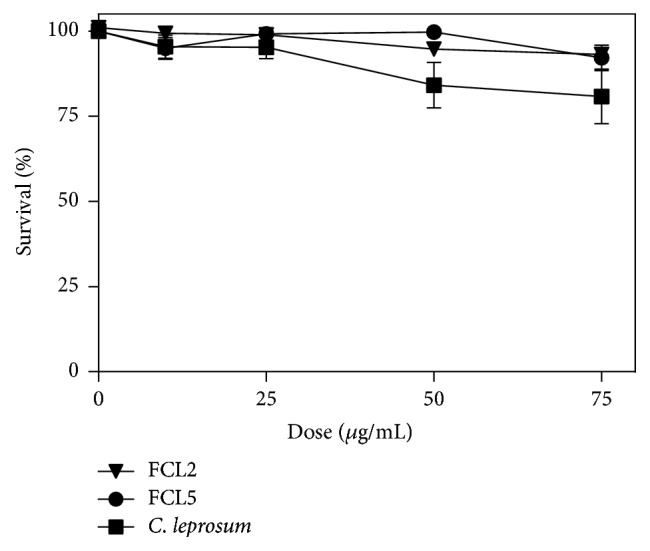
Survival of V79 cells after exposure to crude ethanolic extract of* Combretum leprosum* and FCL2 (5,3′-dihydroxy-3,7,4′-trimethoxyflavone) and FCL5 (5,3′,4′-trihydroxy-3,7-dimethoxyflavone) isolated products.

**Figure 3:**
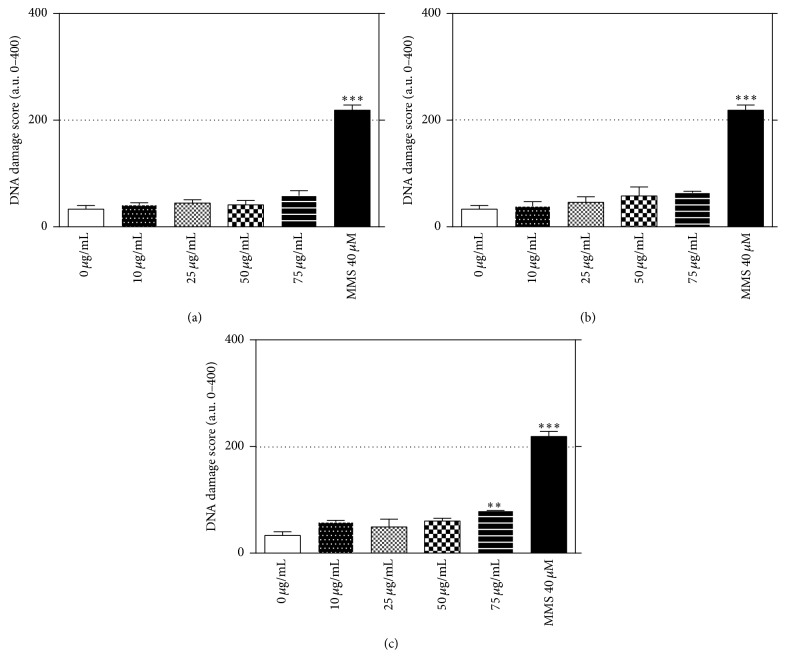
Induction of DNA strand breaks by 3 h treatment with crude ethanolic extract of* Combretum leprosum* (a) and FCL2 (b) and FCL5 (c) products in V79 cells evaluated by Comet Assay. Mean ± SD of three independent experiments. Significantly different in relation to control damage level; ^*∗∗*^ *P* < 0.01; ^*∗∗∗*^ *P* < 0.001.

In V79 cells, the toxicity of these compounds increases from FCL2 = FCL5 < EE (Figure 2). MMS was used as positive control and its cytotoxicity was 56.44%  ± 3.19, as evaluated by MTT assay. Surprisingly, despite the absence of cytotoxicity, the FCL5 flavonoid significantly increased the DNA damage index at 75 *μ*g/mL in these cells [*F*(7.03) = 86.56; *P* < 0.01] (Figure 3). Czeczot et al. [48] tried to estimate the relationship between the positions of hydroxyl and methoxyl groups and the mutagenic activity of some flavonoids using Ames assay. They concluded that the mutagenicity of flavonoids depends on a free hydroxyl or methoxyl group in the* para* 3′ and* ortho* 4′ positions of the B-ring, in which the presence of the methoxy group in the B-ring of the flavonoid molecule markedly decreases the mutagenic activity of the compounds. In this sense, Brown [53] showed that the mutagenicity of various flavonoids could be related to the presence of free hydroxyl groups in the 3′ and 4′ positions of the phenyl ring.

## 4. Conclusion

Natural products have been used in popular medicine to treat several diseases without much knowledge about how harmful these compounds may be to human health. In this work, we showed that the EE of* C. leprosum* presented higher toxicity and mutation induction in the yeast* S. cerevisiae* in comparison with its isolated products (FCL2 and FCL5). The EE also induced the highest cytotoxicity in mammalian V79 cells. Therefore, our results suggest that the antioxidant activity observed for the EE from flowers of* C. leprosum* could be attributed to the presence of flavonoids such as FCL2 and FCL5. Furthermore, the lower cytotoxicity and genotoxicity of these isolated products, especially of FCL2, make them more suitable for pharmacological use.

## Figures and Tables

**Table 1:** *Saccharomyces cerevisiae* strains used in this study.

Strain	Genotype	Enzymatic defense lacking	Source
EG103 (SOD-WT)	*MATα: leu2*Δ*0 his3-*Δ*1 trp1-289 ura3-52*	None	E. Gralla^a^
EG118 (*sod1*Δ)	*Like EG103, except sod1::URA3*	Cu-Zn SOD (cytosolic)	E. Gralla^a^
EG110 (*sod2*Δ)	*Like EG103, except sod2::TRP1*	Mn SOD (mitochondrial)	E. Gralla^a^
EG133 (*sod1*Δ*sod2*Δ)	*Like EG103, except sod1::URA3 and sod2::TRP1*	Both SODs	E. Gralla^a^
XV185-14c (WT)	*MATα: ade2-2 his1-798 lys1-1 trp5-48 hom3-10 arg4-17*	None	von Borstel et al. (1971)^b^

^a^Department of Chemistry and Biochemistry, University of California, Los Angeles, CA 90024-1569, USA.

^b^Reference [54].

**Table 2:** Cytotoxicity and antioxidant effect of *C. leprosum *ethanolic extract in *S. cerevisiae*.

Treatment	Yeast strains			
	WT	*sod1*Δ	*sod2*Δ	*sod1*Δ*sod2*Δ
NC^a^	100.0 ± 0.0	100.0 ± 0.0	100.0 ± 0.0	100.0 ± 0.0
*C. leprosum *10 *μ*g/mL	99.33 ± 2.52	89.60 ± 0.95	100.97 ± 3.65	96.00 ± 3.61
*C. leprosum *50 *μ*g/mL	99.67 ± 4.73	80.93 ± 3.66	98.57 ± 0.67	94.27 ± 3.95
*C. leprosum *100 *μ*g/mL	94.63 ± 4.46	76.43 ± 10.64	94.53 ± 4.17	90.67 ± 0.71
*C. leprosum *500 *μ*g/mL	87.23 ± 2.25	67.50 ± 2.12	96.83 ± 3.33	89.63 ± 1.05
PC^b^: H_2_O_2_ 5 mM	63.03 ± 4.12	16.27 ± 4.88	19.10 ± 4.03	19.33 ± 2.10
*C. leprosum *10 *μ*g/mL + H_2_O_2_	68.53 ± 1.40	28.3 ± 6.65	31.45 ± 2.19	25.70 ± 2.97
*C. leprosum *50 *μ*g/mL + H_2_O_2_	68.33 ± 7.16	43.85 ± 2.90^*∗∗∗*^	38.35 ± 2.19^*∗∗*^	41.25 ± 1.91^*∗∗∗*^
*C. leprosum *100 *μ*g/mL + H_2_O_2_	70.80 ± 2.21	41.65 ± 2.76^*∗∗∗*^	41.50 ± 3.96^*∗∗∗*^	46.70 ± 4.10^*∗∗∗*^
*C. leprosum *500 *μ*g/mL + H_2_O_2_	71.40 ± 5.94	34.90 ± 5.66^*∗∗*^	38.0 ± 7.49^*∗∗*^	35.30 ± 6.51^*∗∗*^

^a^NC: negative control (solvent: DMSO).

^b^Positive control (H_2_O_2_). Data significant in relation to oxidant-treated samples at ^*∗∗*^ *P* < 0.01 and ^*∗∗∗*^ *P* < 0.001/one-way ANOVA, Tukey's multiple comparison test.

**Table 3 tab3:** Cytotoxicity and antioxidant effect of FCL2 product isolated from *C. leprosum *ethanolic extract in *S. cerevisiae*.

Treatment	Yeast strains			
	WT	*sod1*Δ	*sod2*Δ	*sod1*Δ*sod2*Δ
NC^a^	100.0 ± 0.0	100.0 ± 0.0	100.0 ± 0.0	100.0 ± 0.0
FCL2 10 *μ*g/mL	97.3 ± 1.98	94.05 ± 6.43	98.80 ± 1.56	96.50 ± 0.85
FCL2 50 *μ*g/mL	98.05 ± 2.62	94.65 ± 3.89	95.10 ± 2.26	96.25 ± 1.91
FCL2 100 *μ*g/mL	96.90 ± 4.24	93.60 ± 4.38	96.40 ± 4.95	97.10 ± 1.13
FCL2 500 *μ*g/mL	93.70 ± 1.13	94.80 ± 1.27	96.05 ± 3.75	97.80 ± 2.97
PC^b^: H_2_O_2_ 5 mM	63.03 ± 4.12	16.27 ± 4.88	19.10 ± 4.03	19.33 ± 2.10
FCL2 10 *μ*g/mL + H_2_O_2_	63.15 ± 10.25	31.60 ± 2.40^*∗*^	39.10 ± 1.13^*∗∗∗*^	33.20 ± 2.95^*∗*^
FCL2 50 *μ*g/mL + H_2_O_2_	68.95 ± 1.06	38.0 ± 2.12^*∗∗*^	42.80 ± 2.97^*∗∗∗*^	66.53 ± 5.75^*∗∗∗*^
FCL2 100 *μ*g/mL + H_2_O_2_	71.15 ± 1.77	41.60 ± 5.52^*∗∗∗*^	44.0 ± 2.55^*∗∗∗*^	75.15 ± 2.90^*∗∗∗*^
FCL2 500 *μ*g/mL + H_2_O_2_	71.10 ± 3.11	35.80 ± 7.50^*∗∗*^	31.35 ± 2.76^*∗∗*^	66.05 ± 7.71^*∗∗∗*^

^a^NC: negative control (solvent: DMSO).

^b^Positive control (H_2_O_2_). Data significant in relation to oxidant-treated samples at ^*∗*^ *P* < 0.05, ^*∗∗*^ *P* < 0.01, and ^*∗∗∗*^ *P* < 0.001/one-way ANOVA, Tukey's multiple comparison test.

**Table 4:** Cytotoxicity and antioxidant effect of FCL5 product isolated from *C. leprosum *ethanolic extract in *S. cerevisiae*.

Treatment	Yeast strains			
	WT	*sod1*Δ	*sod2*Δ	*sod1*Δ*sod2*Δ
NC^a^	100.0 ± 0.0	100.0 ± 0.0	100.0 ± 0.0	100.0 ± 0.0
FCL5 10 *μ*g/mL	99.65 ± 3.32	99.35 ± 0.78	98.20 ± 1.98	93.75 ± 5.44
FCL5 50 *μ*g/mL	98.15 ± 2.33	99.40 ± 3.82	94.0 ± 5.37	92.20 ± 4.95
FCL5 100 *μ*g/mL	95.05 ± 0.64	96.80 ± 1.56	93.35 ± 4.88	92.25 ± 0.64
FCL5 500 *μ*g/mL	97.25 ± 3.61	93.65 ± 1.63	87.55 ± 1.20	83.30 ± 3.68
PC^b^: H_2_O_2_ 5 mM	63.03 ± 4.12	16.27 ± 4.88	19.10 ± 4.03	19.33 ± 2.10
FCL5 10 *μ*g/mL + H_2_O_2_	68.05 ± 3.04	29.90 ± 0.28^*∗∗*^	38.30 ± 9.33^*∗*^	35.20 ± 2.40^*∗∗*^
FCL5 50 *μ*g/mL + H_2_O_2_	70.55 ± 2.48	36.70 ± 4.53^*∗∗∗*^	43.80 ± 1.41^*∗∗*^	45.10 ± 3.96^*∗∗∗*^
FCL5 100 *μ*g/mL + H_2_O_2_	69.50 ± 5.52	42.35 ± 3.04^*∗∗∗*^	42.20 ± 4.38^*∗∗*^	51.25 ± 6.29^*∗∗∗*^
FCL5 500 *μ*g/mL + H_2_O_2_	68.15 ± 3.75	29.50 ± 1.70^*∗∗*^	35.25 ± 5.16^*∗*^	29.85 ± 1.49^*∗*^

^a^NC: negative control (solvent: DMSO).

^b^Positive control (H_2_O_2_). Data significant in relation to oxidant-treated samples at ^*∗*^ *P* < 0.05, ^*∗∗*^ *P* < 0.01, and ^*∗∗∗*^ *P* < 0.001/one-way ANOVA, Tukey's multiple comparison test.

**Table 5:** Induction of reversion of point mutation for his1-798, ochre allele lys1-1, and *frameshift* mutation (hom3-10) in *S. cerevisiae* haploid strain XV185-14c after treatment with crude ethanolic extract of *C. leprosum*.

Agent	Treatment (*μ*g/mL)	Survival (%)	LYS1/10^8^ survivors^b^	HIS1/10^7^ survivors^a^	HOM3/10^8^ survivors^a^
STAT cells treated in PBS					
NC^d^	0	100.00	4.00 ± 2.83^c^	10.50 ± 0.71^c^	4.00 ± 1.41^c^
4-NQO^e^	1.0 *μ*g/mL	39.97^*∗∗∗*^	20.85 ± 2.48^*∗∗∗*^	49.50 ± 9.19^*∗∗∗*^	13.50 ± 4.95^*∗*^
*C. leprosum*	10 *μ*g/mL	94.70	7.50 ± 0.71	12.25 ± 0.25	4.00 ± 2.83
	50 *μ*g/mL	91.73	8.00 ± 1.41	12.50 ± 0.71	5.50 ± 2.12
	100 *μ*g/mL	91.73	7.50 ± 2.12	12 ± 2.83	5.00 ± 2.83
	500 *μ*g/mL	88.33	10.50 ± 2.12^*∗*^	27.00 ± 7.07^*∗*^	6.00 ± 2.83

^a^Locus-specific revertants. ^b^Locus nonspecific revertants. ^c^Mean and standard deviation per three independent experiments. ^d^Negative control (solvent). ^e^Positive control. Data significant in relation to negative control group (solvent) at ^*∗*^ *P* < 0.05; ^*∗∗∗*^ *P* < 0.001/one-way ANOVA, Tukey's multiple comparison test.

**Table 6:** Induction of reversion of point mutation for his1-798, ochre allele lys1-1, and *frameshift* mutation (hom3-10) in *S. cerevisiae* haploid strain XV185-14c after treatment with FCL2 isolated product from ethanolic extract of *C. leprosum*.

Agent	Treatment (*μ*g/mL)	Survival (%)	LYS1/10^8^ survivors^b^	HIS1/10^7^ survivors^a^	HOM3/10^8^ survivors^a^
STAT cells treated in PBS					
NC^d^	0	100.00	4.00 ± 2.83^c^	10.50 ± 0.71^c^	4.0 ± 1.41^c^
4-NQO^e^	1.0 *μ*g/mL	39.97^*∗∗∗*^	20.85 ± 2.48^*∗∗∗*^	49.50 ± 9.19^*∗∗∗*^	13.50 ± 4.95^*∗∗*^
FCL2	10 *μ*g/mL	98.00	4.00 ± 1.41	10.25 ± 1.77	6.00 ± 1.42
	50 *μ*g/mL	93.03	5.50 ± 2.12	13.15 ± 1.20	5.00 ± 2.83
	100 *μ*g/mL	81.70	7.50 ± 2.12	10.20 ± 4.24	5.00 ± 1.44
	500 *μ*g/mL	82.50	8.50 ± 0.71	10.65 ± 6.29	3.50 ± 2.12

^a^Locus-specific revertants. ^b^Locus nonspecific revertants. ^c^Mean and standard deviation per three independent experiments. ^d^Negative control (solvent). ^e^Positive control. Data significant in relation to negative control group (solvent) at ^*∗∗*^ *P* < 0.01; ^*∗∗∗*^ *P* < 0.001/one-way ANOVA, Tukey's multiple comparison test.

**Table 7:** Induction of reversion of point mutation for his1-798, ochre allele lys1-1, and *frameshift* mutation (hom3-10) in *S. cerevisiae* haploid strain XV185-14c after treatment with FCL5 isolated product from ethanolic extract of *C. leprosum*.

Agent	Treatment (*μ*g/mL)	Survival (%)	LYS1/10^8^ survivors^b^	HIS1/10^7^ survivors^a^	HOM3/10^8^ survivors^a^
STAT cells treated in PBS					
NC^d^	0	100.00	4.00 ± 2.83^c^	10.50 ± 0.71^c^	4.0 ± 1.41^c^
4-NQO^e^	1.0 *μ*g/mL	39.97^*∗∗∗*^	20.85 ± 2.48^*∗∗∗*^	49.50 ± 9.19^*∗∗∗*^	13.50 ± 4.95^*∗∗*^
FCL5	10 *μ*g/mL	89.60	3.27 ± 2.13	7.49 ± 0.71	4.46 ± 3.17
	50 *μ*g/mL	85.67	6.94 ± 0.55	10.24 ± 2.10	4.78 ± 1.90
	100 *μ*g/mL	92.70	6.20 ± 0.29	7.50 ± 0.87	5.10 ± 0.36
	500 *μ*g/mL	76.57	5.95 ± 0.21	9.52 ± 3.10	7.00 ± 1.27

^a^Locus-specific revertants. ^b^Locus nonspecific revertants. ^c^Mean and standard deviation per three independent experiments. ^d^Negative control (solvent). ^e^Positive control. Data significant in relation to negative control group (solvent) at ^*∗∗*^ *P* < 0.01; ^*∗∗∗*^ *P* < 0.001/one-way ANOVA, Tukey's multiple comparison test.

## References

[1] Li J., Wuliji O., Li W., Jiang Z.-G., Ghanbari H. A. (2013). Oxidative stress and neurodegenerative disorders. *International Journal of Molecular Sciences*.

[2] Rahal A., Kumar A., Singh V. (2014). Oxidative stress, prooxidants, and antioxidants: the interplay. *BioMed Research International*.

[3] Bauer G. (2014). Targeting extracellular ROS signaling of tumor cells. *Anticancer Research*.

[4] Verma A. R., Vijayakumar M., Mathela C. S., Rao C. V. (2009). *In vitro* and *in vivo* antioxidant properties of different fractions of *Moringa oleifera* leaves. *Food and Chemical Toxicology*.

[5] Carocho M., Ferreira I. C. (2013). A review on antioxidants, prooxidants and related controversy: natural and synthetic compounds, screening and analysis methodologies and future perspectives. *Food and Chemical Toxicology*.

[6] Atindehou K. K., Schmid C., Brun R., Koné M. W., Traore D. (2004). Antitrypanosomal and antiplasmodial activity of medicinal plants from Côte d'Ivoire. *Journal of Ethnopharmacology*.

[7] Muthu C., Ayyanar M., Raja N., Ignacimuthu S. (2006). Medicinal plants used by traditional healers in Kancheepuram District of Tamil Nadu, India. *Journal of Ethnobiology and Ethnomedicine*.

[8] Gansané A., Sanon S., Ouattara L. P. (2010). Antiplasmodial activity and toxicity of crude extracts from alternatives parts of plants widely used for the treatment of malaria in Burkina Faso: contribution for their preservation. *Parasitology Research*.

[9] Dawe A., Pierre S., Tsala D. E., Habtemariam S. (2013). Phytochemical constituents of *Combretum Loefl*. (*Combretaceae*). *Pharmaceutical Crops*.

[10] Pietrovski E. F., Rosa K. A., Facundo V. A., Rios K., Marques M. C. A., Santos A. R. S. (2006). Antinociceptive properties of the ethanolic extract and of the triterpene 3*β*,6*β*,16*β*-trihidroxilup-20(29)-ene obtained from the flowers of *Combretum leprosum* in mice. *Pharmacology Biochemistry and Behavior*.

[11] De Morais Lima G. R., De Sales I. R. P., Filho M. R. D. C. (2012). Bioactivities of the genus Combretum (*Combretaceae*): a review. *Molecules*.

[12] Eloff J. N., Katerere D. R., McGaw L. J. (2008). The biological activity and chemistry of the southern African Combretaceae. *Journal of Ethnopharmacology*.

[13] Ribeiro S. S., de Jesus A. M., dos Anjos C. S. (2012). Evaluation of the cytotoxic activity of some Brazilian medicinal plants. *Planta Medica*.

[14] McGaw L. J., Rabe T., Sparg S. G., Jäger A. K., Eloff J. N., Van Staden J. (2001). An investigation on the biological activity of Combretum species. *Journal of Ethnopharmacology*.

[15] Lopes L. S., Marques R. B., Pereira S. S. (2010). Antinociceptive effect on mice of the hydroalcoholic fraction and (-) epicatechin obtained from *Combretum leprosum* Mart & Eich. *Brazilian Journal of Medical and Biological Research*.

[16] Longhi-Balbinot D. T., Martins D. F., Lanznaster D., Silva M. D., Facundo V. A., Santos A. R. S. (2011). Further analyses of mechanisms underlying the antinociceptive effect of the triterpene 3*β*, 6*β*, 16*β*-trihydroxylup-20(29)-ene in mice. *European Journal of Pharmacology*.

[17] Longhi-Balbinot D. T., Lanznaster D., Baggio C. H. (2012). Anti-inflammatory effect of triterpene 3*β*, 6*β*, 16*β*-trihydroxylup-20(29)-ene obtained from Combretum leprosum Mart & Eich in mice. *Journal of Ethnopharmacology*.

[18] Ouedraogo M., Baudoux T., Stévigny C. (2012). Review of current and ‘omics’ methods for assessing the toxicity (genotoxicity, teratogenicity and nephrotoxicity) of herbal medicines and mushrooms. *Journal of Ethnopharmacology*.

[19] Viau C. M., Moura D. J., Facundo V. A., Saffi J. (2014). The natural triterpene 3*β*,6*β*,16*β*-trihydroxy-lup-20(29)-ene obtained from the flowers of Combretum leprosum induces apoptosis in MCF-7 breast cancer cells. *BMC Complementary and Alternative Medicine*.

[20] Facundo V. A., Rios K. A., Moreira L. S. (2008). Two new cycloartanes from *Combretum leprosum* Mart. (Combretaceae). *Revista Latinoamericana de Química*.

[21] Burke D., Dawson T., Stearns T. (2000). *Methods in Yeast Genetics: A CSH Laboratory Course Manual*.

[22] Longo V. D., Gralla E. B., Valentine J. S. (1996). Superoxide dismutase activity is essential for stationary phase survival in Saccharomyces cerevisiae: mitochondrial production of toxic oxygen species in vivo. *Journal of Biological Chemistry*.

[23] Cyrne L., Martins L., Fernandes L., Marinho H. S. (2003). Regulation of antioxidant enzymes gene expression in the yeast Saccharomyces cerevisiae during stationary phase. *Free Radical Biology and Medicine*.

[24] Da Costa Júnior J. S., De Almeida A. A. C., Costa J. P., Das Graças Lopes Citó A. M., Saffi J., De Freitas R. M. (2012). Superoxide dismutase and catalase activities in rat hippocampus pretreated with garcinielliptone FC from Platonia insignis. *Pharmaceutical Biology*.

[25] Schuller R. C., von Borstel R. C. (1974). Spontaneous mutability in yeast. I. Stability of lysine reversion rates to variation of adenine concentration. *Mutation Research/Fundamental and Molecular Mechanisms of Mutagenesis*.

[26] Denizot F., Lang R. (1986). Rapid colorimetric assay for cell growth and survival. Modifications to the tetrazolium dye procedure giving improved sensitivity and reliability. *Journal of Immunological Methods*.

[27] Singh N. P., McCoy M. T., Tice R. R., Schneider E. L. (1988). A simple technique for quantitation of low levels of DNA damage in individual cells. *Experimental Cell Research*.

[28] Nadin S. B., Vargas-Roig L. M., Ciocca D. R. (2001). A silver staining method for single-cell gel assay. *Journal of Histochemistry and Cytochemistry*.

[29] Collins A. R., Ai-guo M., Duthie S. J. (1995). The kinetics of repair of oxidative DNA damage (strand breaks and oxidised pyrimidines) in human cells. *Mutation Research-DNA Repair*.

[30] Burlinson B., Tice R. R., Speit G. (2007). Fourth international workgroup on genotoxicity testing: results of the in vivo Comet assay workgroup. *Mutation Research—Genetic Toxicology and Environmental Mutagenesis*.

[31] Williams R. J., Spencer J. P. E., Rice-Evans C. (2004). Flavonoids: antioxidants or signalling molecules?. *Free Radical Biology and Medicine*.

[32] Halliwell B. (2007). Dietary polyphenols: good, bad, or indifferent for your health?. *Cardiovascular Research*.

[33] Halliwell B. (2009). The wanderings of a free radical. *Free Radical Biology and Medicine*.

[34] Tang S. Y., Halliwell B. (2010). Medicinal plants and antioxidants: what do we learn from cell culture and *Caenorhabditis elegans* studies?. *Biochemical and Biophysical Research Communications*.

[35] Gutteridge J. M. C., Halliwell B. (2010). Antioxidants: molecules, medicines, and myths. *Biochemical and Biophysical Research Communications*.

[36] Vasconcellos M. C., Moura D. J., Rosa R. M. (2010). Evaluation of the cytotoxic and antimutagenic effects of biflorin, an antitumor 1,4 o-naphthoquinone isolated from *Capraria biflora* L. *Archives of Toxicology*.

[37] Jamieson D. J. (1998). Oxidative stress responses of the yeast *Saccharomyces cerevisiae*. *Yeast*.

[38] Dizdaroglu M., Kirkali G., Jaruga P. (2008). Formamidopyrimidines in DNA: mechanisms of formation, repair, and biological effects. *Free Radical Biology and Medicine*.

[39] Pannangpetch P., Taejarernwiriyakul O., Kongyingyoes B. (2008). P-145 Ethanolic extract of *Combretum decandrum* Roxb. decreases blood glucose level and oxidative damage in streptozotocin-induced diabetic rats. *Diabetes Research and Clinical Practice*.

[40] Gouveia M. G. S., Xavier M. A., Barreto A. S. (2011). Antioxidant, antinociceptive, and anti-inflammatory properties of the ethanolic extract of combretum duarteanum in rodents. *Journal of Medicinal Food*.

[41] Liochev S. I., Fridovich I. (2007). The effects of superoxide dismutase on H_2_O_2_ formation. *Free Radical Biology and Medicine*.

[42] De Freitas J. M., Liba A., Meneghini R., Valentine J. S., Gralla E. B. (2000). Yeast lacking Cu-Zn superoxide dismutase show altered iron homeostasis: role of oxidative stress in iron metabolism. *Journal of Biological Chemistry*.

[43] Rice-Evans C. (2004). Flavonoids and isoflavones: absorption, metabolism, and bioactivity. *Free Radical Biology and Medicine*.

[44] Rice-Evans C. A., Miller N. J., Paganga G. (1996). Structure-antioxidant activity relationships of flavonoids and phenolic acids. *Free Radical Biology & Medicine*.

[45] Pietta P.-G. (2000). Flavonoids as antioxidants. *Journal of Natural Products*.

[46] Van Acker S. A. B. E., van den Berg D.-J., Tromp M. N. J. L. (1996). Structural aspects of antioxidant activity of flavonoids. *Free Radical Biology and Medicine*.

[47] Dueñas M., González-Manzano S., González-Paramás A., Santos-Buelga C. (2010). Antioxidant evaluation of O-methylated metabolites of catechin, epicatechin and quercetin. *Journal of Pharmaceutical and Biomedical Analysis*.

[48] Czeczot H., Tudek B., Kusztelak J. (1990). Isolation and studies of the mutagenic activity in the Ames test of flavonoids naturally occurring in medical herbs. *Mutation Research/Genetic Toxicology*.

[49] Tyagi A. K., Agarwal C., Singh R. P., Shroyer K. R., Glode L. M., Agarwal R. (2003). Silibinin down-regulates survivin protein and mRNA expression and causes caspases activation and apoptosis in human bladder transitional-cell papilloma RT4 cells. *Biochemical and Biophysical Research Communications*.

[50] De Naeyer A., Vanden Berghe W., Pocock V., Milligan S., Haegeman G., De Keukeleire D. (2004). Estrogenic and anticarcinogenic properties of kurarinone, a lavandulyl flavanone from the roots of Sophora flavescens. *Journal of Natural Products*.

[51] Dhanalakshmi S., Agarwal C., Singh R. P., Agarwal R. (2005). Silibinin up-regulates DNA-protein kinase-dependent p53 activation to enhance UVB-induced apoptosis in mouse epithelial JB6 cells. *Journal of Biological Chemistry*.

[52] Pereira B. K., Rosa R. M., Silva J. D. (2009). Protective effects of three extracts from Antarctic plants against ultraviolet radiation in several biological models. *Journal of Photochemistry and Photobiology B: Biology*.

[53] Brown J. P. (1980). A review of the genetic effects of naturally occurring flavonoids, anthraquinones and related compounds. *Mutation Research/Reviews in Genetic Toxicology*.

[54] von Borstel R. C., Cain K. T., Steinberg C. M. (1971). Inheritance of spontaneous mutability in yeast. *Genetics*.

